# A Simple Rule for Estimating the Amount of Solids in the Urine

**Published:** 1914-01

**Authors:** 


A Simple Rule for Estimating the Amount of Solids in the Urine.—Dr. L. Duncan Bulkley, says: "Multiply the last two figures of the specific gravity of the urine by the number of ounces voided in twenty-four hours, and add 10 per cent to the product. Thus, if the amount passed in twenty-four hours was 36

ounces, and the specific gravity 1.021, it would be $36\times21=756+10$ per cent=831, the number of grains of solids in the whole amount. By comparing this with the table it can readily be ascertained if the amount is above or below the normal standard for the body weight of any patient." He says that the method is Haines' modification of Hasser's.—*Journal of Cutaneous and Genito-Urinary Diseases.*

